# A Herbal Prescription of Insamyangyeongtang as a Therapeutic Agent for Frailty in Elderly: A Narrative Review

**DOI:** 10.3390/nu16050721

**Published:** 2024-03-01

**Authors:** Han-Gyul Lee, Ichiro Arai, Seungwon Kwon

**Affiliations:** 1 Department of Cardiology and Neurology, Kyung Hee University College of Korean Medicine, Kyung Hee University Medical Center, Seoul 02447, Republic of Korea; gyulee0614@hanmail.net; 2 Graduate School of Pharmaceutical Sciences, Nihon Pharmaceutical University, Tokyo 113-0034, Japan; i-arai@nichiyaku.ac.jp

**Keywords:** Insamyangyeongtang, Ninjin’yoeito, frailty, tonifying qi and blood, elderly

## Abstract

Frailty is a major geriatric syndrome with a multifactorial etiology that induces a decline in multiple physiological and psychological functions. In traditional East Asian medicine (TEAM), qi and blood deficiency clinically represent as fatigue, anemia, anorexia, decreased strength after illness, and weakness, commonly interpretated as frailty. An herbal prescription of Insamyangyeongtang (IYT, Ninjin’yoeito in Japanese, Ren-Shen-Yang-Rong-Tang in Chinese) tonifies qi and blood and has the potential to treat multiple targets caused by qi and blood deficiency. As the population ages and frailty increases, there is an increase in the potential effectiveness of IYT in frailty. This study reviewed relevant clinical trials to provide an updated view on the effect of IYT on frailty. IYT has therapeutic effects on frailty associated with chronic respiratory diseases (e.g., chronic obstructive pulmonary disease) and cognitive impairments (e.g., Alzheimer’s disease) and improves respiratory symptoms and cognition. IYT also has therapeutic effects on weight gain, muscle mass, and strength, and improves nutritional status in frail elderly individuals who have decreased muscle mass and strength, loss of appetite, and weight loss. The same effect has been shown in frailty in elderly individuals with rehabilitation treatment and chronic diseases. IYT also improves frailty associated with symptoms such as intractable dizziness and genitourinary symptoms. The beneficial effects of IYT in several diseases could be important for medication replacement, reduction, and prevention of polypharmacy. Based on the results of this review, we suggest that IYT has the potential to be a therapeutic agent against frailty.

## 1. Introduction

Frailty is a common geriatric syndrome that is characterized by age-related decline in physiological reserve and function of multiorgan systems. It increases vulnerability to negative health outcomes [1], resulting in a huge burden on medical and social systems [2,3]. As human lifespan has increased since the 1950s, the proportion of the elderly population has rapidly increased [4], and an awareness of the need to prevent or delay the onset of frailty is expanding globally [2,5]. Aging, genetics, lifestyle, diseases, and environment are some of the etiological factors of frailty. However, much remains to be discovered about the complex multifactorial etiology of this syndrome [1]. The current pharmacologic approaches used for the treatment and prevention of frailty are either not based on evidence or have significant adverse effects [6,7], and the implementation in clinical use requires further investigation [8]. Therefore, only exercise and the prevention of stressors through comprehensive geriatric interdisciplinary interventions have emerged as the best treatments for frailty at the present [1].

Traditional East Asian medicine (TEAM) is a traditional medicine developed in China and other East Asian countries such as Korea and Japan. TEAM is based on several ancient philosophical theories of the East Asian region, including Yin and Yang, the five elements, the meridian system, Zang-fu, essence, spirit, qi, blood, body fluid, and pattern identification [9,10]. In these, qi corresponds to vital energy, while blood is the basic substance for the growth and development of human organs, skin, muscles, nerves, and bones [11]. TEAM has traditionally considered the deficiency of qi or/and blood to be the cause of degenerative diseases or aging [12,13]. Qi deficiency is defined as a lack of power in the whole body, inducing dysfunction in various parts of the body and is often caused by aging, overwork, and chronic illness [14]. Blood deficiency is a pathological condition of blood dysfunction and organ dystrophy, similar to anemia, and is caused by massive blood loss, anemia, insufficient hematopoiesis, and blood stasis [13]. Therefore, the deficiency of qi and blood is clinically quite similar to frailty, which refers to the overall deterioration of the structure and function of the human body as it ages.

Among the herbal prescriptions used as therapeutics by TEAM, numerous herbal prescriptions have been used to tonify qi and blood deficiency. Insamyangyeongtang (IYT, Ninjinyoeito in Japanese, or Ren-Shen-Yang-Rong-Tang in Chinese) is one of the herbal prescriptions with tonifying effects [15] and has been used to improve symptoms of anemia, anorexia, cough, fatigue, and facilitate disease recovery [16]. Recently, IYT has attracted significant attention in diseases of elderly patients, such as cancer, chronic diseases, cognitive impairments, respiratory diseases, psychiatric disorders, and frailty [17]. Particularly, the versatility of IYT suggests its potential as a therapeutic agent for frailty (which has a complex multifactorial etiology). This review aimed to provide the scientific background and reports related to the practical applicability of IYT in the treatment of frailty.

## 2. Scientific Studies on the Herbal Medicine Prescription of IYT

### 2.1. The Origin of the Tonification Effect of IYT

IYT was first recorded in the classic Chinese medical reports titled ‘Prescriptions from the Great Peace Imperial Grace Pharmacy’ (1151, Taipinghuiminhejijufang in Chinese, Taepyeonghyeminhwajegukbang in Korea, and Taiheikeiminwazaikyokuhouin Japanese). IYT consists of 12 herbal medicines: *Panax ginseng*, *Astragalus membranaceus*, *Atractylodis Rhizoma Alba*, *Wolfiporia extensa*, *Glycyrrhiza uralensis*, *Angelica gigas*, *Paeonia lactiflora* Pall., *Rehmanniae Radix* Preparata, *Cinnamomum verum*, *Citri unshius Pericarpium*, *Polygala tenuifolia*, and *Schisandra chinensis*. *Panax ginseng* and *Astragalus membranaceus* are herbal medicines that are known to tonify qi, and *Angelica gigas*, *Paeonia lactiflora* Pall, and *Rehmanniae Radix* Preparata to tonify blood. Therefore, IYT tonifies both qi and blood. IYT contains many physiologically active substances and functions that can improve symptoms and aid recovery from diseases [18]. Herbal prescriptions are composed of several herbs according to ancient concepts and have been applied to treat various diseases and disorders in modern medicine. IYT is widely used in modern applications to treat qi and blood deficiency. In Japan, the national health insurance system covers the conditions of decreased physical strength after recovery from illness, fatigue, loss of appetite, night sweats, cold feet, anemia, poor complexion, persistent cough, insomnia, mental imbalance, physical fitness after illness or childbirth, and weak constitution [19]. Recently, the clinical effectiveness of IYT in treating frailty in gastrointestinal, respiratory, and urinary functions has attracted attention [18].

### 2.2. Effect of IYT on Frailty Associated with Chronic Respiratory Diseases

Chronic respiratory diseases are closely related to frailty [20]. In particular, chronic obstructive pulmonary disease (COPD) is the most frequently studied chronic respiratory disease associated with frailty [21]. Reports suggest that COPD doubles the risk of frailty [22]. Frailty is also common in elderly individuals with idiopathic pulmonary fibrosis (IPF) [23]. IPF is a progressive, chronic respiratory disease that occurs primarily in elderly individuals and causes early, activity-limiting dyspnea, weakness, and fatigue [24]. Previous research suggests that administration of IYT for respiratory diseases might be feasible [18]. *Schisandra chinensis*, one of the herbal constituents of IYT, is commonly used to treat respiratory diseases and symptoms of cough, asthma, and sputum [25]. This increases the possibility of applying IYT to frailty with chronic respiratory diseases.

First, IYT may be beneficial for respiratory-related and emotional function in patients with COPD and frailty. Hirai et al. conducted a randomized controlled trial [26] and reported the effects of IYT in patients with COPD and frailty. Sixty-two patients with COPD were enrolled in that study. The patients were randomly divided into an IYT (dried extract 6.7 g/day) group (*n* = 31) and a control (standard treatment) group (*n* = 31). The primary outcome was changes in Kihon checklist (KCL) score, which revealed changes in frailty. The secondary outcome was changes in Simplified Nutritional Appetite Questionnaire (SNAQ), COPD Assessment Test (CAT), and Hospital Anxiety and Depression Scale (HADS) at week 24. There was a difference in the changes in the KCL scores between the IYT and control groups, but the difference was not statistically significant (*p* = 0.09). However, there were statistically significant improvements in the SNAQ (*p* = 0.03), CAT (*p* = 0.03), HADS-Anxiety (*p* < 0.01), and HADS-Depression (*p* = 0.02) scores in the IYT group compared with the control group. These results suggest that IYT could be an effective treatment with diverse effects on patients with COPD and frailty, despite being administered standard treatment.

IYT is also useful in improving fatigue in patients with chronic respiratory diseases. In a retrospective study, the effect of IYT on patients with interstitial pneumonia (IP) and fatigue was reported [27]. Sixteen patients with IP were treated with IYT dried extract (6.7 g/day) for 12 weeks. The primary outcome was the Chalder Fatigue Scale (CFS) score; secondary endpoints were the SNAQ and modified Medical Research Council (mMRC) scores. The results showed a significant decrease in CFS scores before and after the IYT (*p* = 0.0389). The SNAQ score did not change (*p* = 0.8145), and the mMRC showed a decrease in median score but the difference was not statistically significant (*p* = 0.0956). In laboratory tests, serum albumin, a serum marker of IP, significantly increased (*p* = 0.0458) and Klebs von den Lungen-6 (KL-6) significantly decreased (*p* = 0.0104). No significant changes in respiratory function or IYT-induced adverse events were observed. Considering the progressive and irreversible disease characteristics of IP, treatment for IP is to maintain and improve the quality of life. These results suggest that IYT may have a therapeutic effect on frailty and quality of life by improving fatigue in patients with IP.

The clinical outcomes of IYT are supported by several case reports. One case reported an improvement in frailty in a patient with severe COPD after IYT therapy [28]. A 76-year-old male patient with COPD was treated with multiple bronchodilators, nutritional therapy, patient education, and respiratory rehabilitation, but continued to have anorexia, weight loss, weakness, decreased physical activity, depression, and anxiety. Improvement in these symptoms began one month after prescribing IYT. Six months after prescribing IYT, the patient’s KCL, CAT, HADS-Anxiety, and HADS-Depression scores were all significantly reduced, and the patient showed marked improvement in frailty with weight and muscle mass gains, resulting in an almost normal health status. In another case series, patients with frailty were hospitalized for COPD or pneumonia [29]. An 87-year-old male was hospitalized for COPD and acute prostatitis. The patient showed sustained improvement in the 36-Item Short Form Health Survey (SF-36) scores after both 4 and 12 weeks of IYT administration. A 65-year-old male patient with COPD and urinary tract infection showed improvement in all SF-36 component scores after 12 weeks of IYT administration. An 80-year-old male patient with pneumonia showed increased SF-36 scores after 4 weeks of IYT administration. Kushima et al. reported the use of IYT for anorexia and lethargy in patients with IPF [30]. A 59-year-old male patient with IPF showed objective improvement in anorexia, fatigue, and weight gain after 12 weeks of treatment with standard therapy combined with IYT. Another 59-year-old male patient was treated with standard therapy plus IYT for 12 weeks and showed no changes in anorexia but showed improvement in fatigue and weight gain. The details of these studies are presented in Table 1.

### 2.3. Effect of IYT on Frailty Associated with Cognitive Impairments

Several studies have demonstrated a significant association between frailty and cognitive impairment or dementia [31,32,33]. Although physical activity such as aerobic training, resistance training, tai chi [34], or a combination of these exercises are thought to be protective against frailty and cognitive impairment [35], there are few interventions for the prevention and treatment of frailty and cognitive impairment [36].

In these circumstances, the potential of IYT as a novel treatment option for cognitive impairment in patients with frailty has been investigated. Previous studies have shown that IYT improves cognitive function by reversing demyelination caused by aging [37], and also improves cognitive function in Alzheimer’s disease (AD) [38]. *Polygala tenuifolia*, one of the constituent herbs of IYT, could enhance cognitive function [39] and has been shown to improve memory function (forgetfulness) [40,41]. Further, IYT might improve dementia based on a constituent herb Gamiondam-tang (Jiaweiwendantang in Chinese, and Kamiuntanto in Japanese), which is a frequently prescribed herbal prescription for neurosis [42]. Therefore, IYT may play a role in preventing and treating frailty in cognitive decline.

In an open-label pilot study, the effects of IYT were evaluated in patients with AD and frailty who complained of anorexia, apathy, and cognitive dysfunction [43]. Twenty patients were treated with IYT (dried extract 4 or 6 g/day) for 12 weeks. The primary outcome was the Neuropsychiatric Inventory (NPI)-anorexia score. The secondary outcomes were the NPI-apathy, vitality index, Mini-Mental State Examination (MMSE) score, and physical and blood nutrition indices, which were assessed at baseline (week 0) and at 4, 8, and 12 weeks. After week 4, the anorexia and apathy scores of the NPI and the amount of dietary intake significantly improved. The vitality index (*p* < 0.05) and MMSE (*p* < 0.001) scores significantly improved at 12 weeks. Five adverse events were reported; however, these were either unrelated to IYT (such as falling) or non-serious (such as vomiting). As anorexia and AD are closely related, frailty, apathy, and cognitive impairment affect each other and exacerbate the disease [33], and frailty and apathy do not usually recover as AD progresses. Therefore, the early treatment of frailty and apathy in patients could be seen as an important factor in the prognosis of the disease. The results of this study suggested the potential of IYT as a novel treatment for cognitive impairment by simultaneously affecting frailty, apathy, and cognitive decline in patients with AD.

In another open label exploratory study, the effectiveness of IYT was analyzed in patients with mild cognitive impairment (MCI) or mild AD plus frailty or prefrailty [44]. Among the 14 patients, 9 had MCI, 5 had mild AD, 11 were frail, and 3 were prefrail. All the patients were administered IYT (dried extract 4 or 6 g/day) for 24 weeks. The primary outcome was the NPI-anorexia score, and the secondary outcomes were the Japanese version of the Cardiovascular Health Study criteria (J-CHS) score, body composition, fatigue visual analogue scale (VAS), blood nutrition index, controlling nutritional status (CONUT) score, total lymphocyte count, total cholesterol level, NPI-depression and apathy, global Clinical Dementia Rating (CDR), CDR-Sum of Boxes (SB) score, and the Japanese version of the Montreal Cognitive Assessment (MoCA-J), which were all evaluated at baseline (week 0), 4, 8, 16, and 24 weeks. The NPI-anorexia scores improved significantly in every evaluation period (4 w, *p* < 0.05; 8 w, *p* < 0.01; 16 w, *p* < 0.001; 24 w, *p* < 0.001). After 24 weeks, the J-CHS score was significantly reduced (*p* < 0.01), and no frailty was observed as all patients returned to the prefrailty or healthy state. In addition, the VAS score for fatigue was significantly reduced at week 16 (*p* < 0.05), and the NPI depression score was significantly reduced at weeks 8 and 16 (*p* < 0.05). No significant changes were observed in body composition or CONUT score. Global CRD, CRS-SB, and MoCA-J scores remained at baseline levels through 24 weeks. The results of that study also suggested that IYT has a multidimensional effect on clinical outcomes.

These multidimensional clinical effects were supported by individual clinical cases. We reviewed a case report of three AD patients with loss of appetite and weight loss treated with IYT [45]. The AD patients (84-year-old female, 74-year-old female, 81-year-old male) were administrated IYT (dried extract 3.35 g/day) for 9 months or 14 months. After IYT administration, body weight, muscle mass, and weight after removing body fluid weight had increased in all three patients.

These results suggest that IYT could be safe and effective in treating frailty, particularly anorexia and fatigue, in MCI and mild AD patients, and may be helpful in the prognosis of cognitive impairment. Details of these reviews are presented in Table 2.

### 2.4. Effect of IYT on Frailty Associated with Aging

The ability to recover from physiological damage declines with aging, and the accumulation of physiological damage increases the risk of frailty in the elderly, such as muscle mass loss, depression, and anorexia [46]. Additionally, frailty in the elderly is closely related to poor nutrition and can cause sarcopenia, which is a decrease in muscle mass [47]. Therefore, appropriate intervention, nutritional therapy [48], and exercise therapy [49] are recommended in elderly patients with frailty. Despite this, the evidence level for these interventions is insufficient. A previous study reported that when administered IYT, most frail patients with qi and blood deficiency regained their health quickly [50]. Therefore, the possibility of IYT frailty among the elderly was predicted.

In a comparative study, the effectiveness of IYT was compared in elderly patients with frailty [51]. A study of 113 elderly patients with at least one of the symptoms for decreased strength after illness (malaise, hypophagia, night sweats, cold hands and feet, or anemia) were included. The patients were divided using an envelope method into the IYT group (*n* = 64) and control group (*n* = 49). The IYT group was administered IYT dried extract (6.7 g/day) and conventional treatment for 24 weeks, and the control group was administered only the conventional treatment. Grip strength and muscle quality scores were used to evaluate the treatment effects. The results showed that the grip strength of the left and right hands of the IYT group improved significantly before and after treatment (both *p* < 0.01) but remained unchanged or decreased in the control group. In the between-group comparison, the IYT group showed significantly improved hand strength on both sides after treatment compared with the control group (right, *p* < 0.01; left, *p* < 0.001). The muscle quality score did not change before or after treatment in the IYT group but decreased significantly in the control group (*p* < 0.05). There were significant differences in the between-group comparisons after treatment (*p* < 0.01). The study suggested that IYT affects the preservation or improvement of muscle strength by activating the protein kinase, which is activated by promoting ghrelin production, stimulating PGC-1α secretion in skeletal muscle, thereby activating muscle mitochondria. Although this study focused on sarcopenia, considering that physical frailty interacts with psychological and social frailty [52], it was suggested that IYT has the potential to treat overall frailty in the elderly population.

A previous study evaluated the effects of IYT on various physical functions regardless of muscle strength. An open-label, non-comparative, prospective, multicenter, post-marketing survey was conducted to investigate the safety and efficacy of IYT in the elderly [46]. This study included elderly outpatients taking IYT for at least one of the following indications: deterioration in constitution after disease, fatigue/malaise, anorexia, night sweats, coldness of limbs, and anemia. Safety was evaluated based on the occurrence of adverse events (including abnormal laboratory values) during IYT intake. Efficacy was assessed by VAS scores for fatigue/malaise and anorexia and the basic checklist by the Ministry of Health, Labor, and Welfare of Japan. All assessments were performed at 0, 8, 16, and 24 weeks. Efficacy was analyzed in 537 patients, and VAS scores significantly decreased in the fatigue/malaise and anorexia groups at 8, 16, and 24 weeks and 8 and 24 weeks, respectively. On the basic checklist, the number of patients expected to require nursing care significantly decreased at 24 weeks compared with baseline in four areas (activities of daily living, motor function, oral function, and depression). In the physician assessment, IYT was evaluated as “effective” or “moderately effective” by 486 of 537 patients (90.5%). Of the 808 patients included in the safety analysis, 31 adverse events occurred in 25 patients, with an incidence rate of 3.1%. Among these, 29 were classified as mild, with the most common adverse events being gastrointestinal disorders (2.1%). This study contributes to the safety data by showing that IYT does not cause serious adverse events. Considering that the basic checklist assesses all domains of frailty [53], it also showed the efficacy of the IYT in improving and preventing not only physical but also psychiatric/psychological frailty in the elderly.

In a retrospective study, the weight maintenance effect of IYT in elderly patients with chronic diseases and decreased appetite and fatigue was evaluated [54]. Eleven elderly patients with chronic respiratory, neurological, and digestive diseases were treated with IYT (dried extract 4 or 6 g/day) for at least 6 months, and their pre- and post-treatment body weight, serum body weight, and serum levels of total protein and albumin were compared. The results showed no significant pre- or post-treatment differences in any of the three categories. It was suggested that *Panax ginseng*, a major constituent of IYT, maintained the nutritional effects in elderly with chronic diseases. Suzuki et al. [46] found that IYT maintained or improved weight in elderly patients with chronic diseases. However, this study examined serum protein and albumin levels, providing more convincing evidence for the nutritional maintenance effect of IYT.

Two case reports reported results consistent with the previous findings. Morinaga et al. [55] reported enhancing rehabilitation and frailty improvement effects of IYT in elderly patients who developed sarcopenia after surgery for hip fracture. A 92-year-old male patient was admitted to a rehabilitation hospital two weeks after surgery for a left femoral neck fracture. IYT (dried extract 6.7 g/day) was prescribed for 2 months, starting 2 weeks after admission, for appetite loss and spinal rehabilitation. Although body weight and body fat percentage decreased, the muscle mass increased. In the nutritional status evaluation, there was an increase in the serum levels of transferrin, prealbumin, and retinol-binding protein, which indicated the amount of food consumed, and the average daily calorie intake also increased from 992 kcal to 1159 kcal. During the rehabilitation evaluation, the FIM score increased from 49 to 105. Sakisaka et al. [56] evaluated bilateral handgrip strength and muscle quality scores in three frail elderly patients with previous cancer or cerebrovascular disease after 24 months of long-term IYT (dried extract 6.7 g/day) treatment. The results showed an increase in grip strength and an improvement in muscle quality score in all three cases. Details of these reviews are presented in Table 3.

### 2.5. Effect of IYT on Frailty Associated with Other Conditions

A retrospective study reported on the effects of IYT in combination with dizziness rehabilitation on dizziness and frailty in patients with chronic intractable dizziness [57]. A total of 31 patients with intractable dizziness were grouped into 14 frailty groups and 17 non-frailty groups based on the presence/absence of frailty. The treatment for dizziness was dizziness rehabilitation consisting of 22 different therapy lessons (seven, eight, three, and four sitting, standing, walking, and supine exercises, respectively) in both groups. The treatment for frailty was IYT dried extract 6.7 g/day in addition to exercise and nutritional therapy. All treatments were performed for 6 months, and dizziness and frailty were assessed using the Dizziness Handicap Inventory (DHI), an evaluation of frailty symptoms, the Kihon checklist, and the VAS of fatigue on the first day of hospitalization and at 1, 3, and 6 months. The results showed that DHI scores for dizziness significantly improved at 1, 3, and 6 months compared to pre-treatment (53.3 ± 19.4 to 37.1 ± 23.9 (*p* < 0.01), 30.84 ± 22.2 (*p* < 0.01), and 28.0 ± 21.9 (*p* < 0.01), respectively). In the frailty assessment, the number of frailty symptoms was significantly reduced at 1, 3, and 6 months compared to pre-treatment (2.4 ± 1.4 to 1.1 ± 1.4 (*p* < 0.01), 0.9 ± 1.2 (*p* < 0.01), and 1.0 ± 1.2 (*p* < 0.01), respectively), and 11 out of 14 patients reached a non-frail state after 6 months of treatment. Kihon checklist also improved significantly at 1, 3, and 6 months compared to pre-treatment (5.1 ± 2.8 to 4.3 ± 2.8 (*p* < 0.05), 3.4 ± 2.2 (*p* < 0.01), and 3.4 ± 2.4 (*p* < 0.01), respectively). There was also a significant decrease in fatigue VAS scores at 3 and 6 months compared to pre-treatment (50.8 ± 27.7 mm to 37.3 ± 28.2 mm (*p* < 0.01) and 28.0 ± 25.8 mm (*p* < 0.01), respectively). Patients with frailty are at risk of falling, which adversely affects their prognosis [58]. This study suggests that the administration of IYT may prevent falls by improving dizziness symptoms.

Genitourinary symptoms, such as overactive bladder, and genitourinary syndromes of menopause in the elderly are known to lead to frailty in the elderly, resulting in a decreased quality of life [59,60]. In a single-center, retrospective cohort study, the effectiveness of IYT was examined in frail female patients aged 65 years or older with genitourinary symptoms [61]. Patients were divided into an IYT group (that included pelvic floor muscle training and IYT administration (dried extract 6.7 g/day)) and a non-IYT group (that included only pelvic floor muscle training). All treatments were performed for one year. The Fatigue, Resistance, Ambulation, Illnesses, and Loss of Weight (FRAIL) scale was used to assess frailty. The International Consultation on Incontinence Questionnaire-Short Form (ICIQ-SF) and Overactive Bladder Symptom Score (OABSS) were used to assess urinary symptoms, and the vaginal health index score (VHIS) and vulvodynia swab tests were used to assess genital symptoms before and after treatment completion. In each group, 159 patients completed the treatment and evaluation. The results showed that the change in FRAIL scale was significantly higher in the IYT group compared to the no-IYT group (0.13 ± 0.37 vs. 0.01 ± 0.10, respectively, *p* = 0.001). Urinary symptoms were significantly improved in the IYT group for both OABSS change (0.89 ± 1.65 vs. 0.36 ± 1.14, respectively, *p* = 0.001) and ICIQ-SF score change (1.51 ± 1.75 vs. 0.42 ± 1.18, respectively, *p* < 0.001). Genital symptoms were significantly better in the IYT group in terms of change in VHIS (0.58 ± 1.08 vs. 0.21 ± 0.65, respectively, *p* < 0.001). Five percent of patients in the IYT group experienced a reduction in the dose of antimuscarinic drugs used to treat the overactive bladder. No significant adverse effects were observed.

These reports demonstrate that IYT improves frailty and chronic symptoms in the elderly not only in respiratory diseases and cognitive impairment, for which there is a large body of evidence, but also in frailty caused by chronic symptoms that may occur in the elderly.

### 2.6. Possible Effect of IYT on Polypharmacy Associated with Frailty

Polypharmacy, or the use of multiple of medications, is classified as a geriatric syndrome and is commonly present in the elderly [62]. Polypharmacy has become an important issue in the elderly as it is associated with falls, functional impairment, adverse drug reactions, hospitalization and readmission, decreased quality of life, and mortality [63,64,65]. Since polypharmacy and frailty are both common and widely studied concepts in older patients, they may have impact one another [66]. A previous study showed that older adults with polypharmacy tend to have significantly higher frailty scores [67]. Another systematic review suggested that reducing polypharmacy in the elderly may be an important way to prevent and treat frailty [68]. Therefore, there is a need for strategies to address polypharmacy in frailty management.

**Figure 1:**
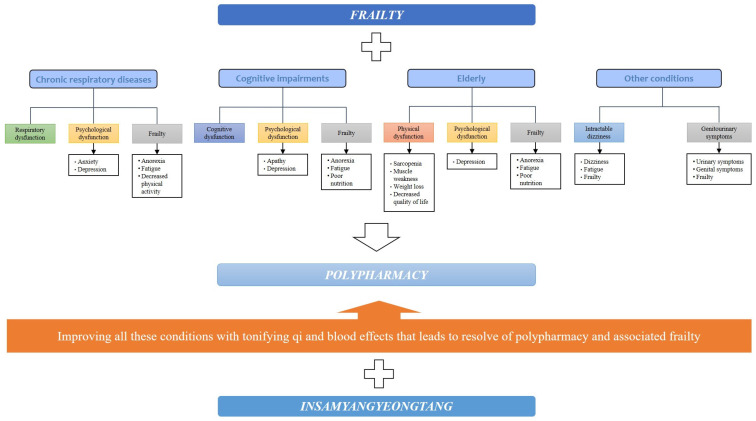
Potential for IYT to be a new candidate for polypharmacy in elderly patients with frailty. IYT: Insamyangyeong-tang.

The prevalence of polypharmacy in the elderly is because as they live longer, they are more likely to experience the coexistence of multiple health problems [69]. Therefore, to reduce polypharmacy, a treatment that has a multifaceted effect on multiple diseases and their comorbidities is needed. IYT has been shown to improve respiratory symptoms in chronic respiratory disease, disease-related frailty, and psychological conditions such as anxiety and depression, resulting in an improved quality of life. In cognitive impairment, IYT has multidimensional effects such as improvement in cognition, anorexia, apathy, fatigue, depression, and nutritional status. Regarding frailty associated with aging, the IYT group showed improvements in muscle strength and body weight, and improved anorexia, fatigue, nutritional status, overall physical function, and psychological status. In addition, there was an improvement in the symptoms and frailty in patients with intractable dizziness and genitourinary symptoms. Taken together, these effects of IYT suggest that IYT may contribute to polypharmacy reduction by replacing some of the effects of conventional medications or may be a preventive method for medication escalation owing to its multifaceted effects on these chronic conditions. For example, in elderly patients with Alzheimer’s disease taking donepezil, choline alfoscerate, appetite stimulants, antidepressants, nutraceuticals, and anticholinergics for cognitive decline, anorexia, depression, fatigue, and overactive bladder symptoms, IYT may replace or reduce the effects of these medications in whole or in part. This process may ultimately lead to the elimination of polypharmacy, thus reducing the risk of polypharmacy. IYT may also improve frailty caused by the polypharmacy itself (Figure 1).

## 3. Conclusions and Clinical Recommendation

Traditionally known for its qi and blood tonification effects, IYT has long been used to treat qi and blood deficiency conditions such as anorexia, fatigue, anemia, weak constitution, and decreased strength after illness. Based on these effects, IYT has recently been used in the treatment of frailty in the elderly. In this study, we reviewed the literature on the effects of IYT on frailty. Evidence to date indicates that IYT could be used as a novel alternative therapy for treating frailty associated with chronic respiratory diseases, cognitive impairments, elderly with chronic diseases or rehabilitation state, and various chronic symptoms, and for treating each of the associated diseases or symptoms. These multifaceted effects of IYT in elderly individuals susceptible to polypharmacy suggests that IYT might eliminate polypharmacy-induced risk and. However, to date, most studies are retrospective or are case reports that do not provide high-level evidence. Furthermore, although some studies have described the pharmacological mechanisms of IYT as modulation of mitochondrial dysfunction or anti-anorexic effects [43], the definitive pharmacological mechanisms remain uncertain. Therefore, high-quality prospective clinical trials and experimental studies to identify detailed pharmacological mechanisms are needed.

## Figures and Tables

**Table 1:** Effect of Insamyangyeong-tang on frailty associated with chronic respiratory disease.

First Author (Year)	Subjects, Design and Intervention	Results
Hirai (2020) [26]	62 patients with COPD with frailty Randomized controlled trial IYT group: IYT (dried extract 6.7 g/day) for 24 weeks (*n* = 31) Control group: Conventional treatment for 24 weeks (*n* = 31) Primary outcome: Changes of KCL score (frailty basic checklist score) Secondary outcomes: Changes of SNAQ, CAT, HADS	Primary outcome: IYT [−1 (−3 to 0)] vs. control [0 (−2 to 0)], *p* = 0.09 SNAQ: IYT [1 (0 to 2)] vs. control [0 (0 to 1)], *p* = 0.03 CAT: IYT [−1 (−4 to 1)] vs. control [1 (−1 to 3)], *p* = 0.03 HADS-Anxeity: IYT [−2 (−3 to 0)] vs. control [1 (−1 to 1)], *p* < 0.01 HADS-Depression: IYT [0 (−3 to 0)] vs. control [0 (−2 to 2)], *p* = 0.02
Kushima (2021) [27]	16 patients with interstitial pneumonia with fatigue Retrospective study IYT (dried extract 6.7 g/day) + conventional treatment for 12 weeks Primary outcome: CFS Secondary outcomes: SNAQ, mMRC	CFS significantly decrease (17.1 ± 6.8 to 13.4 ± 5.7, *p* = 0.0389) SNAQ increased without significance (14.3 ± 1.6 to 14.4 ± 1.9, *p* = 0.8145) mMRC median decreased without significance [3 (2–3) to 2 (1–3), *p* = 0.0956]
Kuniaki (2018) [28]	A 76-year-old patient with severe COPD with frailty Case report IYT (dried extract 6.7 g/day) for 1 month	Body composition (kg): Body weight (46.5 to 54.5), Muscle mass (32.1 to 38.3). Factors: CAT (26 to 12), KCL (15 to 6), HADS-Anxiety (13 to 5), HADS-Depression (15 to 5)
Kashima (2021) [29]	3 frail patients who started taking IYT from the time of discharge after being hospitalized for COPD and pneumonia Case report IYT (dried extract 6.7 g/day) for 12 weeks SF-36 component: Physical function, PF; role physical, RP; body pain, BP; general health, GH; vitality, VT; social function, SF; role emotional, RE; mental health, MH	Case 1 (87-year-old male with COPD): SF-36 score 0, 4, 12 weeks after taking IYT [(BP; 41, 51, 100), (SF; 50, 100, 100), (MH; 75, 75, 90) Case 2 (65-year-old male with COPD): SF-36 score 0, 12 weeks after taking IYT [(PF; 70, 95), (RP; 75, 100), (BP; 72, 84), (GH; 45, 52), (VT; 37.5, 75), (RE; 75, 100), (MH; 70, 90) Case 3 (80-year-old with Pneumonia): SF-36 score 0, 4 weeks after taking IYT [(RP; 68.8, 100), (BP; 52, 61), (GH; 52, 72), (VT; 43.8, 62.5), (SF; 37.5, 100), (RE; 58.3, 91.7)
Kushima (2021) [30]	2 patients with idiopathic pulmonary fibrosis Case report IYT (dried extract 6.7 g/day) for 12 weeks	Case 1 (59-year-old male): SNAQ (15 to 16), CFS (6 to 4), Body weight (51.8 kg to 55.2 kg) Case 2 (59-year-old male): SNAQ (14 to 14), CFS (27 to 24), Body weight (50.2 kg to 51.9 kg)

Abbreviations: COPD, Chronic obstructive pulmonary disease; IYT, Insamyangyeong-tang; KCL, Kihon checklist; SNAQ, Simplified Nutritional Appetite Questionnaire; CAT, COPD Assessment Test; HADS, Hospital Anxiety and Depression Scale; CFS, Chalder Fatigue Scale; mMRC, modified Medical Research Council; SF-36, 36-Item Short Form Health Survey.

**Table 2:** Effect of Insamyangyeong-tang on frailty associated with cognitive impairments.

First Author (Year)	Subjects, Design and Intervention	Results
Ohsawa (2017) [43]	20 frail AD patients with anorexia and apathy Single-arm, nonrandomized, historical-controlled study IYT (dried extract 4 or 6 g/day) for 12 weeks Primary outcome: NPI-anorexia score Secondary outcomes: NPI-apathy score, vitality index, MMSE score, physical and blood nutrition indices	NPI-anorexia score significantly decreased by week 4 (baseline: 4.85 ± 0.58; 4 w: 3.06 ± 0.60, *p* < 0.05; 8 w: 1.50 ± 0.43, *p* < 0.001; 12 w: 1.00 ± 0.44, *p* < 0.001). NPI-apathy score significantly decreased by week 4 (baseline 5.85 ± 0.65; 4 w: 4.00 ± 0.57, *p* < 0.001; 8 w: 3.38 ± 0.54, *p* < 0.001; 12 w: 3.31 ± 0.51, *p* < 0.001) Meal ingestion amount improved by week 4 (4 w: 0.61 ± 0.14, *p* < 0.01; 8 w: 0.94 ± 0.17, *p* < 0.001; 12 w: 0.94 ± 0.19, *p* < 0.001) Vitality index improved in 12 weeks (baseline: 7.05 ± 0.43; 12 w: 7.94 ± 0.39, *p* < 0.05) MMSE improved in 12 weeks (baseline: 17.32 ± 1.29, 12 w: 19.44 ± 1.30, *p* < 0.001)
Okahara (2023) [44]	14 frail or prefrail patients with MCI (*n* = 9) or mild AD (*n* = 5) A open label exploratory study IYT (dried extract 4 or 6 g/day) for 24 weeks Primary outcomes: NPI-anorexia score Secondary outcomes: J-CHS score, body composition, fatigue VAS, blood nutrition index, CONUT score, total lymphocyte count, total cholesterol level, NPI-depression and apathy, global CDR and CDR-SB, MoCA-J	NPI-anorexia score significantly decreased in 4, 8, 16 and 24 week [Baseline: 8.0 (4.0–8.0); 4 w: 0.0 (0.0–2.8), *p* < 0.01; 8 w: 0.0 (0.0–1.8), *p* < 0.01; 16 w: 0.0 (0.0–0.8), *p* < 0.001; 24 w: 0.0 (0.0–1.0), *p* < 0.01] J-CHS score significantly decreased in 24 weeks [baseline: 3.0 (3.0–3.0); 24 weeks: 2.0 (1.0–2.0), *p* < 0.01] Fatigue VAS score decreased significantly in 16 weeks [baseline: 40.5 (30.0–51.5); 16 weeks: 25.0 (20.3–29.5), *p* < 0.05] NPI-depression score significantly decreased in 8 and 16 weeks [baseline: 1.0 (0.0–1.0); 8 w: 0.0 (0.0–0.8), *p* < 0.05; 16 w: 0.0 (0.0–0.0), *p* < 0.05] No significant change in body composition and CONUT score Global CDR, CDR-SB and MoCA-J scores remained at the baseline level for 24 weeks
Matsui (2021) [45]	3 AD patients with loss of appetite and weight loss Case report Case 1: 84-year-old female, IYT (dried extract 3.35 g/day) for 9 months Case 2: 74-year-old female, IYT (dried extract 3.35 g/day) for 14 months Case 3: 81-year-old male, IYT (dried extract 3.35 g/day) for 14 months	Case 1: Body weight, muscle mass and weight after removing the body fluid weight increased [1.0 kg (50.4 kg to 51.4 kg), 0.6 kg (30.2 kg to 30.8kg), 0.6 kg (31.3 kg to 31.9 kg), respectively] Case 2: Body weight, muscle mass and weight after removing the body fluid weight increased [3.4 kg (53.3 kg to 56.7 kg), 0.4 kg (32.6 kg to 33.0 kg), 3.9 kg (28.8 kg to 32.7 kg), respectively] Case 3: Body weight, muscle mass and weight after removing the body fluid weight increased [2.5 kg (46.8 kg to 49.3 kg), 2.4 kg (34.3 kg to 36.7 kg), 1.0 kg (25.1 kg to 26.1 kg), respectively]

Abbreviations: AD, Alzheimer’s disease; IYT, Insamyangyeong-tang; NPI, Neuropsychiatric Inventory; MMSE, Mini-Mental State Examination; MCI, Mild cognitive impairment; J-CHS, Japanese version of the Cardiovascular Health Study criteria; VAS, visual analog scale; CONUT, controlling nutritional status; CDR, Clinical Dementia Rating; CDR-SB, Clinical Dementia Rating-Sum of Boxes; MoCA-J, Japanese version of the Montreal Cognitive Assessment.

**Table 3:** Effect of Insamyangyeong-tang on frailty associated with elderly.

First Author (Year)	Subjects, Design and Intervention	Results
Sakisaka (2018) [51]	113 elderly frail patients Comparative study using envelop method IYT group: IYT (dried extract 6.7 g/day) + conventional treatment for 24 weeks (*n* = 64) Control group: Conventional treatment for 24 w (*n* = 49) Outcomes: Grip strength, muscle quality score	Grip strength: Significantly improved in IYT group [Right hand: baseline (18.2 ± 5.7) vs. 24 w (19.0 ± 5.8), *p* < 0.01; Left hand: baseline (17.9 ± 5.8) vs. 24 w (19.0 ± 5.8), *p* < 0.01] and vs. control group by amount of changes in 24w [Right hand: IYT group (0.80 ± 2.67) vs. control group (−0.43 ± 2.16), *p* < 0.01; Left hand: IYT group (1.10 ± 3.15) vs. control group (−1.03 ± 2.82), *p* < 0.001] Mucle quality score: No change in IYT group, significantly deteriorated in control group [baseline (42.6 ± 16.0) vs. 24 w (40.6 ± 16.5), *p* < 0.05], compared by amount of change in 24 w, significantly improved in IYT group [IYT group (0.80 ± 2.67) vs. control croup (−0.43 ± 2.16), *p* < 0.01]
Suzuki (2019) [46]	808 elderly patients prescribed IYT in 383 centers Open-label, non-comparative, prospective, multicenter, post-marketing survey Outcomes: Safety (Adverse reaction), Efficacy (VAS scores in fatigue/malaise and anorexia at 8, 16, 24 weeks, Basic Checklist by the Ministry of Health, Labor and Welfare of Japan at 8, 16 and 24 weeks)	① Safety (*n* = 808) Adverse reactions: 31 occurred in 25 patients (Incidence of 3.1%) ② Efficacy (*n* = 537) VAS score: Significantly decreased in fatigue/malaise (8, 16 and 24 weeks, *p* < 0.01) and anorexia (8 weeks, *p* < 0.05; 24 weeks, *p* < 0.01) Basic checklist: The proportion of patients expected to requre nursin care significantly decreased in four domains (activities of daily living, motor function, oral function, and depression) Physician assessment: Related as “effective” or “moderately effective” in 486/537 cases (90.5%)
Nakagawa (2022) [54]	11 elderly patients with chronic wasting disease A retrospective study IYT (dried extract 4 or 6 g/day) for 6 or more months Outcomes: Body weight, serum levels of total protein and albumin before and after IYT administration	Body weight: No significant difference (median: 47.9 kg, range: 43.5–70.0 kg, vs. median: 48 kg, range: 42.7–70.0 kg; *p* = 0.176) Serum total protein level: No significant differnce (median: 7.0 g/dL, range: 5.7–8.2 g/dL vs. median: 6.9 g/dL, range: 5.8–7.9 g/dL; *p* = 0.766) Serum albumin level: No significant differnce (median: 3.9 g/dL, range: 2.8–4.6 g/dL vs. median: 3.9 g/dL, range: 2.4–4.6 g/dL; *p* = 0.550)
Morinaga (2020) [55]	A 92-year-old male patient with hip fracture and sarcopenia Case report IYT (dried extract 6.7 g/day) for 2 months Outcomes: Body weight, body fat percentage, muscle mass, serum level of proteins, total FIM score, average daily calorie intake	Body weight: 61 kg to 56.5 kg Body fat percentage: 34.1% to 21.1% Muscle mass: 38.2 kg to 38.9 kg Serum level of proteins: Total protein (6.8 g/dL to 7.1 g/dL), transferrin (227 mg/dL to 238 mg/dL), prealbumin (24.7 mg/dL to 27.9 mg/dL), retinol-binding protein (2.9 mg/dL to 3.6 mg/dL) Total FIM score: 49 to 105 Average daily calorie intake: 992 kcal to 1159 kcal
Sakisaka (2022) [56]	3 elderly with frailty Case report IYT (dried extract 6.7 g/day) for 24 months Outcomes: Both grip strength, muscle quality score	Case 1 (82-year-old man with prior surgery for lung cancer): Grip strength increased [Right 1.4% (22.0 kg to 22.6 kg), Left 16.9% (16.0 kg to 21.4 kg)], muscle quality score improved 17.1% (35 to 47) Case 2 (68-year-old woman with history of pancreatic cancer): Grip strength increased [Right 3.0% (24.09 kg to 25.55 kg), Left 1.7% (22.30 kg to 23.05 kg)], muscle quality score improved 1.2% (42 to 43) Case 3 (73-year-old woman with sequelae of a cerebral hemorrhage): Grip strength increased [Right 24.5% (9.10 kg to 13.55 kg), Left 0.1% (21.20 kg to 21.25 kg)], muscle quality score improved 0.4% (40 to 40)

Abbreviations: IYT, Insamyangyeong-tang; w, week; VAS, visual analog scale; FIM, functional independence measure.
